# Development of the Lymphoma Enterprise Architecture Database: A caBIG(tm) Silver level compliant System

**DOI:** 10.4137/cin.s940

**Published:** 2009-04-03

**Authors:** Taoying Huang, Pareen J. Shenoy, Rajni Sinha, Michael Graiser, Kevin W. Bumpers, Christopher R. Flowers

**Affiliations:** Winship Cancer Institute, School of Medicine, Emory University, Atlanta, GA, U.S.A

**Keywords:** biomedical informatics grid, non-Hodgkin’s lymphoma, large linked database, semantic integration, caCORE SDK

## Abstract

Lymphomas are the fifth most common cancer in United States with numerous histological subtypes. Integrating existing clinical information on lymphoma patients provides a platform for understanding biological variability in presentation and treatment response and aids development of novel therapies. We developed a cancer Biomedical Informatics Grid™ (caBIG™) Silver level compliant lymphoma database, called the Lymphoma Enterprise Architecture Data-system™ (LEAD™), which integrates the pathology, pharmacy, laboratory, cancer registry, clinical trials, and clinical data from institutional databases. We utilized the Cancer Common Ontological Representation Environment Software Development Kit (caCORE SDK) provided by National Cancer Institute’s Center for Bioinformatics to establish the LEAD™ platform for data management. The caCORE SDK generated system utilizes an n-tier architecture with open Application Programming Interfaces, controlled vocabularies, and registered metadata to achieve semantic integration across multiple cancer databases. We demonstrated that the data elements and structures within LEAD™ could be used to manage clinical research data from phase 1 clinical trials, cohort studies, and registry data from the Surveillance Epidemiology and End Results database. This work provides a clear example of how semantic technologies from caBIG™ can be applied to support a wide range of clinical and research tasks, and integrate data from disparate systems into a single architecture. This illustrates the central importance of caBIG™ to the management of clinical and biological data.

## Background

Lymphomas are a heterogeneous group of cancers that are characterized by abnormal growth of tissue in the lymphatic system. These disorders originate from B-lymphocytes, T-lymphocytes, and natural killer (NK) cells. Between 1950 and 1999, the incidence of Non-Hodgkin’s Lymphoma (NHL) increased by 90% in the United States,^1^ resulting in one of the largest documented increases in cancer. This rapid increase may be a result of improved diagnostic techniques and access to medical care, the rise in HIV-related NHLs, or other causes. Currently, NHL represents approximately 4% of all cancer diagnoses, being the fifth most common cancer among men and women.^2^ In 2008, 66,120 new cases of NHL and 8,220 new cases of Hodgkin lymphoma (HL) are expected to be diagnosed in the United States.^2^

The World Health Organization (WHO) classification system divides lymphomas according to the cell of origin (B, T/NK) and incorporates morphology, immunophenotype, genetic, and clinical features to define subtypes. Approximately 85% of all NHLs are of B-cell origin and the remaining 15% of T-cell origin.^3^ The WHO classification schema for NHL was devised to help aid in prognosis and treatment decision making. The most frequent clinical entities recognized by the WHO classification are diffuse large B-cell lymphoma (DLBCL, 31%) and follicular lymphoma (FL, 22%). The WHO classification divides HL into 2 main types: classical and lymphocyte-predominant (Table 1). Classical HL accounts for 95% of the cases and is further divided into 4 subtypes: nodular sclerosis, mixed cellularity, lymphocyte-depleted, and lymphocyte-rich. Current treatment modalities for lymphoma include conventional chemotherapy, immunotherapy, radioimmunotherapy, and stem cell transplant. Prognostic factors such as age, performance status, and number of relapses can influence how a patient will respond to certain treatments.

In order to expedite the development of innovative clinical and therapeutic strategies for lymphoma, our oncology informatics group has been developing means to integrate existing clinical information into database systems that support cancer research.^4^ To this end, we designed a platform for integrated clinical and biomedical informatics research using patient-level data linking the institution’s existing clinical trials, cancer registry, clinical, administrative, and pharmacy systems with biological databases. However, semantic integration of data from disparate systems remains challenging even when similar concepts are represented in different data systems.

## The Cancer Biomedical Informatics Grid

The cancer Biomedical Informatics Grid (caBIG™)^5^,^6^ is a voluntary network or grid linking individuals and institutions to promote the sharing of data and tools. The caBIG™ development and research covers clinical trials management systems, tissue banks, pathology tools, integrated cancer research, system architecture, vocabularies, common data elements, data sharing, and intellectual capital. The infrastructure and tools established by caBIG™ are likely also to have broad utility outside the cancer community. Currently, more than 900 individuals from over 50 National Cancer Institute (NCI)-designated cancer centers and a multitude of other organizations are working collaboratively on over 70 projects as part of the caBIG™ initiative.^5^,^7^

The systems developed within the caBIG™ community can be organized into four levels of maturity based on different degrees of interoperability defined by the caBIG™ Compatibility Guidelines: Legacy, Bronze, Silver, and Gold.^7^,^8^ Legacy compliance implies no interoperability with an external system or resource. In order to achieve Bronze compatibility, the resource should provide at least programmatic access to data through a public, documented application programming interfaces (API). Silver compatibility requires more conditions, which must provide well-documented API that is based upon an object-oriented abstraction of the underlying data. Gold compatibility includes a service–oriented data and analytical service grid with standardized service advertising and discovery features, and grid–level security strategy. Table 2 details the pertinent categories that must be addressed to obtain caBIG™ Silver compliance: 1) programming and messaging interfaces; 2) vocabularies, terminologies, and ontologies; 3) data elements; and 4) information models (shown in Table 2).^7^

The caBIG™ is creating a common, extensible informatics platform that can integrate diverse data types and support interoperable analytic tools in areas including clinical trials management, tissue banks and pathology, imaging, and integrative cancer research. Table 3 displays the various tools, infrastructure, and data resources in caBIG™ and their roles.

The NCI’s Center for Biomedical Informatics and Information Technology (NCIBIIT) has developed a set of software packages to support application development for cancer research, the caCORE Software Development Kit (SDK).^9^ This SDK provides a platform for data management and semantic integration.

## The Cancer Common Ontological Representation Environment SDK

To establish a common representation within this SDK, the cancer Common Ontological Representation Environment (caCORE) was established to provide a framework for developing syntactically and semantically interoperable biomedical information services. It has several key components: the Enterprise Vocabulary Services (EVS), the cancer Data Standards Repository (caDSR), the Cancer Bioinformatics Infrastructure Objects, and the Common Security Module. A brief description for these components is included in Table 4. Complete documentation and updated information on caCORE and its components can be found on the NCI Center for Bioinformatics (NCICB) web site.

A caCORE SDK generated system has two characteristics: 1) a Model Driven Architecture that provides a conceptual framework and standards for expressing the model, relationships between models, and transformations between models using the Meta-Object Facility, Unified Modeling Language (UML), XML Metadata Interchange (XMI), and Common Warehouse Meta-model specifications, and 2) an n-tier architecture with open API. When a caCORE SDK generated system is combined with controlled vocabularies and registered metadata, the resulting system is semantically integrated with all exposed API elements having runtime accessible metadata that defines the meaning of the data elements using a controlled terminology.

The NCICB has developed the EVS to supply controlled vocabularies, and the caDSR to provide a dynamic metadata registry^10^ specifically for cancer informatics applications. Systems developed using the caCORE methodology use the same approach for defining, registering, and adopting data and representation of standards. Clients of those systems can therefore extract information from multiple data sources using similar API calls, and can rely upon the semantic equivalence of the data retrieved.

## Semantic Structure of caBIG™

One of the problems confronting the biomedical data management community is the vast number of ways that similar or identical concepts are described. Such inconsistency in data descriptors (metadata) makes it challenging to aggregate and manage even modest-sized data sets and share data across current information resources. Consider for instance, the number of different ways that each of the 51 types of lymphoma shown in Table 1 can be coded if one or more is represented in various administrative, clinical, pathological, radiology, or clinical trials databases. Examples of different coding schemes include International Classification of Diseases, Tenth Revision (ICD-10) diagnosis codes,^11^ International Classification of Diseases for Oncology (ICD-O) topography and histology codes,^12^ and institutional representations within clinical trials systems that may be based on the WHO classification system^13^ or the older Working Formulation, Kiel, or Revised European-American Lymphoma classification systems.^14^

In order to address these problems, the NCI created the EVS, which forms the semantic underpinnings of caCORE. Semantic interoperability lies in the UML model, the use of publicly accessible terminologies/vocabularies/ontologies (EVS-NCI Thesaurus) and the use of publicly accessible metadata repository (caDSR). The EVS organizes distinct but overlapping terminologies and thus provides a rich controlled vocabulary for data coding and retrieval including the NCI Thesaurus and the NCI Metathesaurus. The controlled terminology component of caBIG™ is maintained in the NCI Thesaurus. The NCI Metathesaurus is based on National Library Medicine’s UML System Metathesaurus supplemented with additional cancer-centric vocabulary. It maps many biomedical vocabularies useful to the cancer community and contains both public domain and proprietary vocabularies.^15^

**Figure 1.**
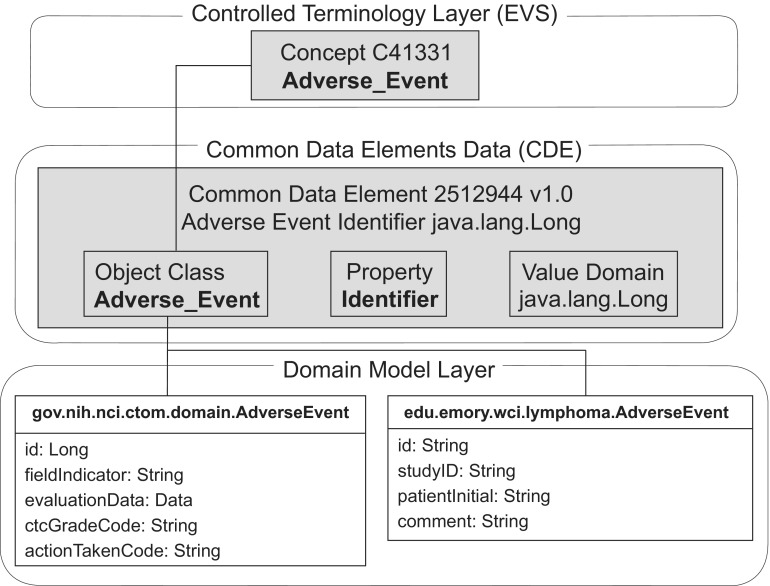
**Layers of semantic interoperability in caBIG**™. Semantic interoperability lies in UML model, use of publicly accessible Terminologies/vocabularies/ontologies (EVS–NCI Thesaurus) and use of publicly accessible metadata repository (caDSR).

The caBIG™ organizes semantic metadata in three layers of abstraction, as illustrated in Figure 1. At the top level, semantic concepts are organized through the NCI Thesaurus, and accessed through the EVS. These concepts are related to each other through the use of Common Data Elements (CDEs) which are stored and accessed through the caDSR. The bottom layer is the Domain Model layer where each UML class is linked to a concept within the NCI Thesaurus, each relationship between UML classes is linked to an association, and each relationship between a UML class and an attribute value is linked to a CDE.

Using the caBIG™ semantic modeling methodology, Tobias et al developed a model by which the College of American Pathologists cancer protocols could be used as the basis for an electronic data standard in pathology.^16^ Wang et al. developed a Lung Cancer Clinical Database Application System using caCORE SDK.^17^,^18^ There are approximately 69 Silver compliant systems registered with the caBIG™.^19^ The models investigate cancer registries, clinical trials, gene expression, genomics, and behavioral research data management.

The data system described herein is the first system that aids lymphoma research and is registered with caBIG™ (http://umlmodelbrowser.nci.nih.gov/umlmodelbrowser/). As of writing of this paper, we are not aware of any other lymphoma databases developed using caCORE SDK. However, there are lymphoma databases developed but not registered with caBIG™. For example, the Lymphoma NCI Specialized Programs of Research Excellence initiated at the University of Iowa/Mayo Clinic is a highly successful lymphoma translational research program.^20^ The Lymphoma Foundation of America is an independent, nonprofit charitable organization that conducts lymphoma research especially on dietary factors, environmental factors, treatment, and genetics.^21^ Other national lymphoma databases include the Surveillance, Epidemiology, and End Result^22^ database and National Cancer Database.^23^

Using the semantic metadata framework (Fig. 1), we have developed a caBIG™ silver compliant database, the Lymphoma Enterprise Architecture Data-system (LEAD™), that establishes domain specific ontologies and meta–data for lymphoma translational research and lymphoma clinical research. With LEAD™ deployed, we have established reusable data structures for institutional case-control studies, national SEER cohort studies, and lymphoid malignancy clinical trials. This work provides a clear example of how semantic technologies from caBIG™ can be applied to support a wide range of clinical and research tasks, and illustrates the central importance of caBIG™ to the management of clinical and biological data.

## Methods

### LEAD™ Development

**Figure 2.**
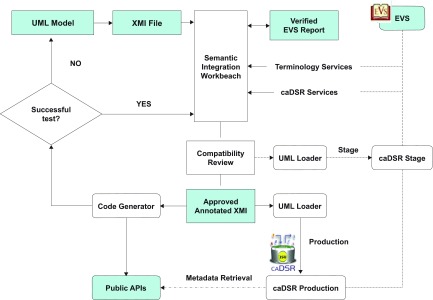
**The caCORE workflow.** This figure describes the steps involved in creating a silver level compliant system. A UML object model is the input into the workflow. The model is exported from the format native to the tool it was developed into the standard XMI representation. The XMI file is then annotated with terminology services. Once the annotated XMI is reviewed and approved, it is used as input to generate code and public APIs, and it is deposited into production caDSR.

**Figure 3.**
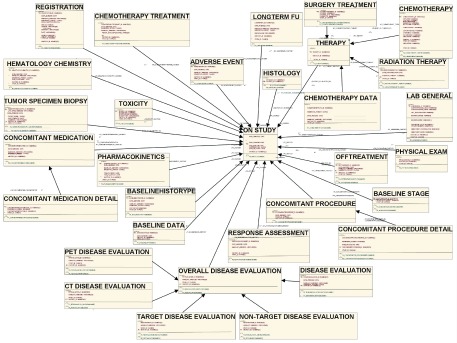
**Logic Model for lymphoma clinical database developed using Enterprise Architecture.** This figure demonstrates the relationships between the key data elements in LEAD™. Components within each key element are not represented in this figure due to practical constraints of resolution and size.

As a member of caBIG™ community, we followed the caCORE SDK guidance and developed LEAD™ in accordance with the Silver compatibility guidelines. Steps involved in caCORE SDK workflow and the development of LEAD™ are shown in Figure 2.^24^ The major steps in the workflow include: using case development; information modeling; semantic annotation; metadata registration; code generation; and system deployment.^25^ LEAD™ development involved creating class diagrams and data models within Enterprise Architecture (EA). The structure and relationships between classes in the LEAD™ model are shown in Figure 3. The actual software code, such as API for data access, data services, is generated from the model.

In the model, classes represent discrete scientific entities. For instance, in LEAD™, a patient’s demographic information is denoted by class Registration, and his/her histological diagnosis is modeled by class Histology. Disparate methods for representing diagnosis (e.g. ICD-9 codes, ICD-O codes, pathology-free text reports) are semantically integrated by mapping to this class. Attributes of each class represent specific characteristics of the entities and become Data Elements in the software system. For example, one of the attributes of Histology is defined as ‘immune_phenotype’. In addition to classes and attributes, the model also specifies the associations between classes including cardinality and direction. For instance, each patient has only one registration record, but he can have multiple adverse events. Thus, the association between class Onstudy and class AdverseEvent is one–to–many relationship. It is important and required that the UML model be annotated with descriptions. This facilitates the subsequent semantic integration. In LEAD™, UML entities are matched to vocabulary concepts; and annotated by an expert in the subject area (CF).

After the UML model was created, the NCI EVS staff were involved the annotation of entities. Once the annotated UML model was approved by the model owner, it was loaded into the caDSR by the NCI EVS staff. The caCORE SDK automatically generates code for web services, an API for data access and a basic class browser. The class browser allows the developer to check the attributes in a class, and to search based on the criteria the user enters. The result set is displayed for all those records that meet the search criteria. Any other classes that have associations with this class are searched and displayed as well. After the data system is deployed biomedical researchers can query the system through well-documented API, web browsers, or web services.

In this manuscript we describe the use of semantic tools to integrate data from heterogeneous systems. LEAD™ compiles data from multiple data different sources with unique data elements including: the ONCORE^®^^26^ data from clinical trials, clinical data from Emory University’s legacy administrative (Health Quest: hospital; IDX: clinic), cancer registry (IMPAC Medical Systems), electronic medical records (Cerner PowerChart), laboratory, pharmacy, clinical trials databases^4^ and data from populated SEER registry. All these data sources have unique representations of data elements that are integrated using caBIG™ semantic tools (Fig. 3). For example, the clinical trials data has information on patient identifiers, clinical and laboratory data, and data on the treatment and response. The Emory University clinical data on the other hand is comprised of linked data from the clinical, administrative, pharmacy and biological databases that contains clinical, laboratory, and treatment response data coded using different schemas and terminology. The SEER data has detailed sociodemographic information in addition to the details on the disease histology, treatments, and survival represented in yet another manner. Through LEAD™ we integrated the data from these data sources into a single comprehensive database with a unified semantic meaning for concepts shared across these databases.

**Figure 4.**
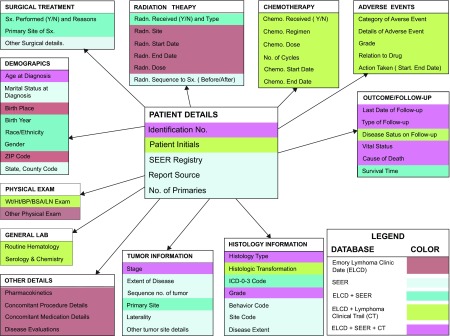
**Relations between entities and classes from all data sources.** This figure depicts the color–coded sources of various key data elements of LEAD™. The codes for colors are shown in the legend. **Abbreviations:** ELCD, Emory Lymphoma Clinical Data; SEER, Surveillance, Epidemiology, and End Result; CT, Emory University Lymphoma Clinical Trials data.

The relationship between entities and classes in the model and data fields from lymphoma cohort studies, SEER registry data, and clinical trials case report forms are shown in Figure 4. The EA model was used to generate an XMI file and Data Definition Language Script. Classes and attributes within the model were iteratively annotated by authors TH, CF, and NCI’s EVS personnel and the final annotated XMI file was uploaded to the caDSR. Once the LEAD™ metadata passed compatibility review, the final APIs were created using the SDK code generator. Development was implemented on and supported by Dell™ PowerEdge™ 1800 web server. Once stabilized, the LEAD™ application was migrated to a Dell™ PowerEdge™ 6800 production server running an Oracle 10 g relational database.

The Semantic Web is a vision for the next generation of the Web.^27^ The first generation Web is characterized by static and handwritten HTML pages; the second generation is characterized by dynamics and interactive HTML pages. However, these two generations share a similar property; the information on Web is represented only in natural language for human processing. The goal of the next generation, namely Semantic Web, is to make information on the Web available for computational processing.

True semantic integration requires a common and shared vocabulary. The ontology serves this purpose and Semantic Web technology provides language for this goal. The World Wide Web consortium approved two key Semantic Web technologies: the Resource Description Framework (RDF) and the Web Ontology Language (OWL). The RDF provides a common data model so that when RDF is built on top of XML, systems can achieve the level of interoperability required by highly dynamic and integrated bioinformatics applications.^28^ Both RDF and OWL are Semantic Web standards that provide a framework for sharing the data on the Web. OWL builds on RDF and RDF Schema, and adds more vocabulary for describing properties and classes. Thus, OWL has strict and precise semantics that are not found in RDF Schema. As Semantic Web resources become mature and available, there is an increasing tendency in bioinformatics applications to use Semantic Web technologies.^29^ This higher level of semantic integration may be possible in future versions of LEAD™.

**Figure 5.**
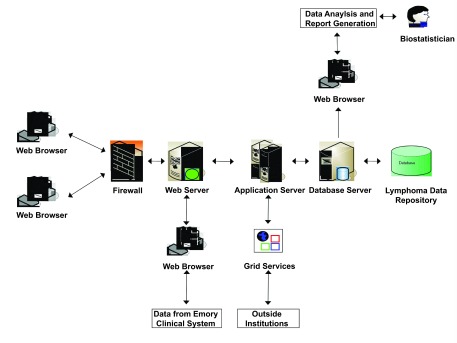
**Architecture for LEAD™.** This figure describes the architecture of the Lymphoma Enterprise Architecture Data–system. It contains the presentation tier, business tier and data source tier. The web server passes the requests from web browsers and transfers them to the application server which then accesses the backend database and generates the required content dynamically and sends the response back to the web browser through the web server. Outside community accesses the data through the provided programmatic API.

To use LEAD™, the researcher interacts with a web browser through the Internet to input and query the relevant information (Fig. 5). The web browser sends the user’s request to the web server, the web server parses the requests and transfers the requests to the application server, the application server accesses the backend database using object-relational mapping, generates the required content dynamically, and sends the response back to the web browser through the web server. In order for the outside cancer research community to access the data stored in the lymphoma clinical database application system, the system provides programmatic APIs generated using caCORE SDK code generator.

### Populating the database with lymphoma data

To populate LEAD™, we utilized data sources from three ongoing research studies: 1) a cohort studies of NHL patients previously treated at Emory University, 2) SEER registry data on patients with lymphoid malignancies, and 3) phase 1 lymphoma clinical trials data.

#### Emory University Clinical Data

Emory University clinical source data for LEAD™ was derived from a large linked database that represents a fully integrated platform combining clinical and administrative legacy databases.^4^ This platform interfaces Emory Healthcare’s existing legacy administrative (Health Quest: hospital; IDX: clinic), cancer registry (IMPAC Medical Systems), electronic medical records (Cerner PowerChart), laboratory, pharmacy, clinical trials databases creating a stand-alone structured query language-(SQL) based data warehouse.

We utilized a series of search strategies to identify a joint population of interest containing potential patients with selected NHL subtypes; FL, DLBCL, and mantle cell lymphoma (MCL). We searched the linked database using cancer registry ICD-O histology codes to identify patients: FL(9690, 9695, 9691, 9698), DLBCL (9680, 9684), and MCL (9693).^30^ The query also included the ICD-O behavior code 3 (malignant neoplasms, primary). This query is labeled Q1 in Table 5A. The next series of queries involved text searches of the electronic medical records using simple free text with the histological diagnosis or more complex free-text including synonyms from the UML System Metathesaurus Concept Search.^4^ Each text string search was conducted twice, once limited to anatomical pathology (AP) reports, and once accessing all medical records. Table 5A describes the phrases used to identify patients with FL, DLBCL, and MCL. A list of 2471 patients was obtained from the text search of AP reports and 3170 patients were identified from the text search of electronic medical records. Each query defines a different schema for representing patients with a lymphoma diagnosis within the clinical data repository.

Next, a group of trained abstractors (PS, KB, and TH) ascertained each patient’s histological diagnosis by reviewing pathology reports and medical charts. In all cases, WHO classification schema for NHL was utilized as the gold standard for diagnosis.^31^ A hematological oncologist (CF) resolved all cases where there was uncertainty as to whether the WHO criteria were met. The pathology-verified diagnosis status for each individual was used to calculate the sensitivity and specificity of each query strategy as follows:

**Table:** 

	Lymphoma Subtype Positive	Lymphoma Subtype Negative	
Query	a	b	Sensitivity
Positive			= a/a + c
Query	c	d	Specificity
Negative			= d/b + d

Individuals identified by AP and medical reports to have a definitive diagnosis of FL, DLBCL, or MCLwere integrated into LEAD™ using the semantic architecture to map individuals identified by the means described in each query into a unified definition of histological subtype. Data elements were mapped into LEAD™ representations using a Perl script and loaded into the LEAD™ Oracle 10 g database.

#### SEER data

The SEER registry is an authoritative source of information on cancer incidence and survival in the United States. The SEER limited-use data include SEER incidence and population data. Eighty-two fields from the SEER dataset have been incorporated into LEAD™. Data were obtained in a tab-delimited format and mapped to LEAD™ data elements.

Next, we built a 2-tier web interface by using Java 2 Enterprise Edition (J2EE) technology to query the data in the relational database. This web interface simplifies reading and searching SEER data. The user interacts with a web interface that uses JSP and a set of rich, custom tag libraries to provide the view. The Java Server Page communicates with the database management system through object-relational mapping. This provides a powerful, flexible platform for allowing users to query SEER lymphoma data. Moreover, the data are stored in a semantic framework such that data analyses examining the SEER lymphoma populations can be readily compared to equivalent populations of Emory patients since they are mapped to the same LEAD™ concept. The generated LEAD™ application system is a distributed, web-based application which provides two interfaces: one is a web-based user interface for inputting and querying data; the second provides programmatic APIs for outside institutions to query the data stored in the backend database using object/relational mapping technology (Fig. 5).

#### Lymphoma clinical trials data

Web forms for the clinical trial database were generated using a web application template developed in J2EE.^32^ This web application is meta-data driven, which means all the requirements that are used to generate a web form are stored in a relational database (e.g. column names, data types, data length, constraints, etc), allowing all of the web forms to be generated dynamically. If a change needs to be made on the form (e.g. converting a text field to a selection list) no programming effort is required. Through modifying the metadata in the database by SQL, the change is reflected immediately when the form is reloaded. The web application template was used to generate data entry forms for database table.

## Results

We performed data integration across disparate data sources: 1) to illustrate integration capabilities of the LEAD™ infrastructure, and 2) to establish datasets that facilitate use by physicians and researchers with a common interface.

### Integration of emory university clinical data for cohort studies

Query strategies varied in terms of their sensitivity and specificity across lymphoma subtypes, but strategies using ICD-O codes had the most favorable characteristics (Table 5B). A total of 930 patients (324 FL, 519 DLBCL, and 87 MCL) were verified by histological diagnosis. Queries based on cancer registry histology codes (Q1) had high specificity for FL and MCL but not for DLBCL. Simple free-text queries of pathology reports (Q2) or all medical records documents (Q4) had a high sensitivity for FL, DLBCL, and MCL. Simple free-text searches of all medical records identified 92% of potential FL cases, 64% of DLBCL and 89% of potential MCL cases but had varying sensitivity and specificity: FL(97%, 13%); DLBCL (55%, 50%); and MCL (44%, 6%) (Table 5B). Queries using additional phrases from the UML System synonym list (Q3, Q5) had higher specificity for FL and DLBCL and higher sensitivity for MCL.

No query strategy had ideal characteristics for all lymphoma histological subtypes, thus use of a combination of strategies, representations, and terminologies is required to best identify a robust patient population for clinical research. To render data gathered using heterogeneous terminologies useful, we integrated the resulting data set into LEAD™ by mapping heterogeneous representations for lymphoma histology into the unified meaning ‘Histology Type’ shown in Figure 4. The semantically integrated database allows for data management and data analysis where histological diagnosis has a unique clinical research meaning in LEAD™ regardless of how it was coded in the source data set.

### Integration of surveillance epidemiology and end results^22^ data

SEER registry data for the years 1973–2005 were extracted from the tab-delimited limited-use data set. The entire database contains more than 3.5 million tumors. Lymphoma patients were identified from this data set using ICD-O, Third Edition codes as described previously.^33^ Cases of lymphoma were identified during this time frame and classified into histological diagnosis categories using the WHO system shown in Table 1. One or more ICD-O codes maps to each histology type.^33^

Eighty–two fields from the SEER dataset were incorporated into LEAD™. Tab-delimited data were translated into clinical concepts using the SEER Data Dictionary (http://seer.cancer.gov/manuals/CD2_popdic.html) and mapped to LEAD™ data elements. Figure 4 shows the relationships between SEER and LEAD™ data elements. The common representation of entities and classes between SEER data and other sources (Fig. 4) and the relations between entities and classes across data sources permit data sharing and analysis of data elements with a common meaning. The architecture described permits comparative analyses of institutional and national datasets that address common clinical problems. We have used the representation schema and semantic integration from LEAD™ to investigate the incidence and outcomes for peripheral T-cell lymphoma.^34^ In this instance, LEAD™ provided a common user interface for researchers to examine data regardless of its original source or representation.

### Integration of Phase 1 clinical trials data

FL is the second most frequent lymphoma subtype worldwide with a rapidly increasing incidence in the Western world. The majority of patients with FL present with advanced disease. For these patients, there is no standard treatment and the clinical course is characterized by a pattern of multiple relapses and remissions and a median survival of 6.2 years.^33^ We have developed an early phase clinical trial investigating a novel combination chemotherapy regimen (bortezomib, rituximab, cyclophosphamide, adriamycin, vincristine, and prednisone) designed to improve outcomes for patients with FL. The primary objectives of this study are:

- To identify the maximal tolerated doses of bortezomib and vincristine when used in this combination
- To estimate the complete response rate associated with this regimen

**Figure 6.**
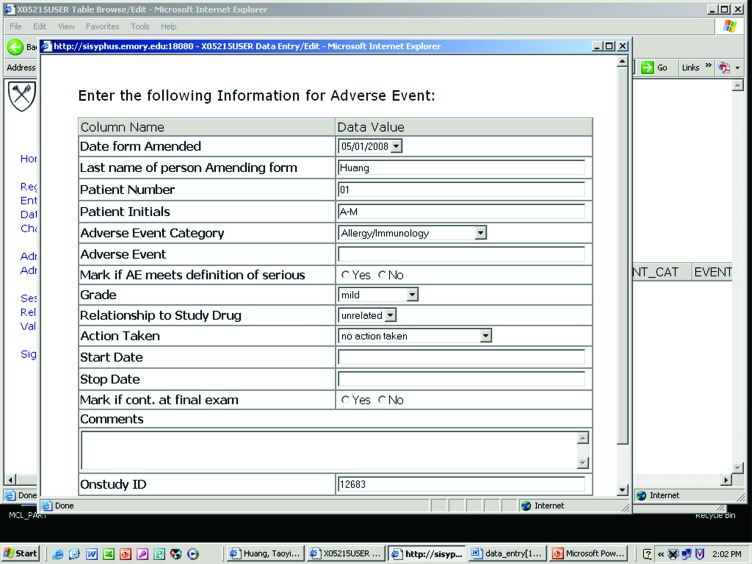
**Graphical user interface for entering adverse event data into LEAD™.** This figure is a sample screen shot of the graphical user interface for entering clinical trial data into LEAD™.

Table 6 shows the schedule for data collections for this lymphoma clinical trial. Figure 6 shows an example graphical user interface for entering clinical trial data into LEAD™. Data collected during the course of the trial are mapped to LEAD™ classes and entities as shown in Figure 4. Again, since LEAD™ relies on the caBIG™ architecture for semantic integration, patients with a histological diagnosis of FL within the clinical trial can be matched to patients who share the same histology in SEER or the Emory lymphoma cohort study. Moreover, since the latter dataset contains patients who overlap with those treated on the clinical trial, data elements that are common to the two studies may be stored once with a common representation and those unique to each study are stored as well with the relationships between the data elements made explicit as shown in Figure 4.

### Semantic queries using LEAD™

Viewing caBIG™ semantic metadata as formal standard ontology, querying can be defined as the search of the members of classes from multiple data sources.^35^ The most important types of queries can be categorized as those that employ joins and merges. If the data services are built upon semantically rich metadata, individual members of different classes ought to be related to each other. Consider for example an individual member of class emory: OnStudy, and an individual of class emory: AdverseEvent, whenever a patient is registered in the clinical trial, this patient has a member of class emory: OnStudy. A query such as the one that seeks to retrieve information about adverse events and event details for a patient, then returns the join on the object property OnStudy has AdverseEvent in class emory: OnStudy and the object property AdverseEvent has AdverseEvent Detail in class emory: AdverseEvent. The strength of caBIG™ semantics in facilitating data integration is obvious, as the join conditions are directly specified by the class properties from the data sources.

The LEAD™ model has been registered in caDSR and can be searched by NCI UML model browser (http://umlmodelbrowser.nci.nih.gov/umlmodelbrowser/). This model can be deployed by another institution to collect and store its lymphoma clinical data. Data stored in this manner will facilitate data integration and could thus promote the conduct of inter-institutional clinical trials and epidemiology studies. Moreover, the model browser permits queries of the model structure and CDEs. An example is querying the adverse events CDEs contained in LEAD™. The execution of this query would return every individual member of AdverseEvent based on the same conditions from these two data sources. It is worth noting that it is necessary to determine one or more data type properties of AdverseEvent, i.e. CDEs which have the class AdverseEvent as its subject that is shared by both subclasses. The researcher who adopts the current LEAD™ UML model can add more attributes or modify the existing attributes in the classes. Readers who are interested in query formulation techniques on semantic data can further reference papers by Shironoshita et al. and Baer et al.^35^,^36^

### Investigational review board approval and controlling access

We obtained Investigational Review Board (IRB) approval for storing and maintaining patient-related data for cancer patients, and an IRB approved process has been developed for incorporating new patient data into our lymphoma database system. This database will use the same procedures for access control that is used for other Emory confidential databases. The research database is protected by the standard Emory Firewall. Dr. Flowers reviews any application for passwords and userIDs from researchers. The Informatics Project Manager (TH) assists investigators in applying for the appropriate level of access, in accordance with the researcher’s individual protocol.

## Discussion

In cancer research, we need to link clinical outcomes and tissue based research data to support the discovery of correlations between molecular studies and prognostic and treatment response profiles. Some of the challenges we face in cancer research include, but not limited to, data sharing, data complexity, and organizational complexity. We also need to use shared languages, such as XML, RDF, or XOL^37^ for reporting. Clinical trials are run over many sites, and data is held by multiple stakeholders and researchers. Thus we need to adopt semantic web technologies, including RDF and OWL to share and integrate data models, which enable us to report results and phenomena with shared languages. Moreover, we should and make it possible to interpret models with more complete and semantically integrated data. The emergence of grid technologies facilitates the collaboration of different centers and allows clinical trials to be scattered in different sites. However, to be meaningful grid technologies must integrate data from multiple stakeholders semantically. Successful semantic integration involves use of controlled vocabularies and structured, standard-based metadata to unambiguously describe diverse data sets.

### Significance

This paper successfully demonstrates the application of caCORE SDK in lymphoma research and this methodology serves as a model for adoption into other cancer research domains, such as lung cancer,^32^ prostate cancer, and breast cancer. The tool sets from the NCI facilitate rapid data modeling, data sharing and exchange, and comprehensive question answering. Hence the tools can expedite drug discovery, cancer detection, and improve disease diagnosis and treatment.

### Limitations and future work

While there has been substantial progress within caBIG™, and in particular in the implementation of caGrid, there is still much work in progress. Although the caCORE SDK provides a rich set of tools to rapidly develop the silver-level compatible system, there is no robust search tool that allows biomedical researchers easily to search the underlying data. Programming effort is still required to extract data from the databases. DBsurfer^38^ is one of such tools that may allow complex queries of semantically integrated databases, but open source tools are needed in this area.

Another issue addressed by the LEAD™ system is the integration of data across numerous legacy systems: Health Quest, IDX, IMPAC Medical cancer registry, Cerner PowerChart electronic medical records, OnCore clinical trials data, and other databases. Other valuable data are contained in different systems, such as data in spreadsheets on personal computers, patient data in electronic systems such as The Emory Electronic Medical Record, and various other cancer database systems that are outside caGrid framework. In the future, caCORE SDK must provide tools for integrating data from legacy systems coded in various formats and performing data quality assurance evaluations. Our future work will concentrate on the provision of grid-enabled access to our lymphoma cancer data and web services within caGrid, through the public APIs, XML, SOAP to allow other data holders to exchange data without intimate knowledge of each other’s system implementations.

## Conclusion

Lymphomas are a heterogeneous group of cancers that require the development of focused research and clinical approaches for specific histological subtypes. Integrating existing clinical, genomic and proteomic information provides a platform for examining biological variability in the pathogenesis of lymphomas and their responses to treatment response and will promote the development of innovative treatment strategies. To expedite the development of novel therapeutics for lymphoma, our oncology informatics group developed a caBIG™ silver-level compliant information system that incorporates clinical and biological data into a semantically integrated structure, LEAD™, that supports information sharing between cancer research scientists and clinicians. The LEAD™ system links cancer registry, pathology, clinical, administrative, pharmacy, and clinical trials data with biological data elements at the patient–level. We demonstrated that the same data elements and structures in LEAD™ could be used for institutional cohort studies linking data across numerous legacy systems,^4^ early phase lymphoma clinical trials data collection, and national SEER registry data on lymphoid malignancies. This caCORE SDK generated system built upon an n–tier architecture with open APIs, utilizes controlled vocabularies and registered metadata to achieve semantic integration. For LEAD™ and other caBIG™ compliant systems the NCI’s EVS supplies the controlled vocabularies, and the caDSR provides dynamic metadata registry. Data from LEAD™ can be combined and reused by systems, programmers, and investigators in the broader cancer community. We have demonstrated that reusable data structures can be applied to a number of research settings and provided examples from institutional retrospective epidemiological studies, national cancer registry data queries, and clinical trials. LEAD™ was populated with data from three ongoing research studies 1) cohort studies of NHL patients previously treated at Emory University, 2) SEER registry data for lymphoma, and 3) case report forms data from phase 1 clinical trials. Source data for each of these research scenarios originated in a variety of formats and, once stored within LEAD™, can be shared and reused based on the standards of the caBIG™ architecture. We have shown how semantic technologies from caBIG™ were applied to support a wide range of clinical and research tasks, and our work serves to illustrate the central importance of caBIG™ to the management of clinical and biological data in cancer research. The infrastructure and tools created by caBIG™ can also benefit researchers outside the cancer community.

## Figures and Tables

**Table 1.** World Health Organization classification for lymphomas.

Non Hodgkin Lymphomas (NHL)	
B-cell Neoplasm, NOS	T-cell and NK-cell Neoplasm, NOS
**Precursor B-cell Neoplasms**	**Precursor T-cell Neoplasm**
– Precursor B lymphoblastic leukemia (9835/3)	– Precursor T lymphoblastic leukemia (9837/3)
– Precursor B lymphoblastic lymphoma (9728/3)	– Precursor T lymphoblastic lymphoma (9729/3)
	– Blastic NK cell lymphoma (9727/3)
**Peripheral (mature) B-cell Neoplasms**	**Peripheral (mature) T-cell Neoplasms**
– Chronic lymphocytic leukemia (9823/3)	– T-cell prolymphocytic leukemia (9834/3)
– Small lymphocytic lymphoma (9670/3)	– T-cell large granular lymphocytic leukemia (9831/3)
– B-cell prolymphocytic leukemia (9833/3)	– Aggressive NK cell leukemia (9948/3)
– Lymphoplasmacytic lymphoma (9671/3)	– Adult T-cell leukemia/lymphoma (9827/3)
– Splenic marginal zone lymphoma (9689/3)	– Extranodal NK/T cell lymphoma, nasal type (9719/3)
– Hairy cell leukemia (9940/3)	– Enteropathy type T-cell lymphoma (9717/3)
– Plasmacytoma/Multiple myeloma (9732/3)	– Hepatosplenic T-cell lymphoma (9716/3)
– Solitary plasmacytoma of bone (9731/3)	– Subcutaneous panniculitis-like T-cell lymphoma (9708/3)
– Extraosseous plasmacytoma (9734/3)	– Mycosis fungoides (9700/3)
– Extranodal marginal zone B-cell lymphoma of mucosa-associated lymphoid tissue (MALT-lymphoma) (9699/3)	– Sezary Syndrome (9701/3)
– Nodal marginal zone B-cell lymphoma (9699/3)	– Primary cutaneous anaplastic large cell lymphoma (9718/3)
– Follicular lymphoma, NOS (9690/3)	– Peripheral T-cell lymphoma, unspecified (9702/3)
– Follicular lymphoma Grade 1 (9690/3)	– Angioimmunoblastic T-cell lymphoma (9705/3)
– Follicular lymphoma Grade 2 (9690/3)	– Anaplastic large cell lymphoma (9714/3)
– Follicular lymphoma Grade 3 (9690/3)	– Lymphomatoid papulosis (9718/1)
– Mantle cell lymphoma (9673/3)	
– Diffuse large B-cell lymphoma (9680/3)	
– Mediastinal (thymic) large cell lymphoma (9679/3)	
– Intravascular large B-cell lymphoma (9680/3)	
– Primary effusion lymphoma (9678/3)	
– Burkitt lymphoma (9687/3)	
– Burkitt lymphoma leukemia (9826/3)	
– B-cell proliferations of uncertain malignant potential	
– Lymphomatoid granulomatosis (9766/1)	
– Post-transplant lymphoproliferative disorder, pleomorphic (9970/1)	
**Hodgkin Lymphoma, NOS**	
– Nodular lymphocyte predominant Hodgkin lymphoma (9659/3)	
– Classical Hodgkin lymphoma (9650/3)	
– Nodular sclerosis classical Hodgkin lymphoma (9663/3)	
– Lymphocyte-rich classical Hodgkin lymphoma (9651/3)	
– Mixed cellularity classical Hodgkin lymphoma (9652/3)	
Lymphocyte-depleted classical Hodgkin lymphoma (9653/3)	

**Source:** Jaffe et al.^31^

**Table 2.** Silver level compatibility guidelines.

Sections	Compatibility Requirements
Programming and Messaging Interfaces	Well–described API approved by the caBIG™ ARCHWS that provide access to data in the form of data objects that are instances of classes represented by a domain model. Electronic data formats reviewed and approved by the caBIG ARCHWS that are supported for both input to and output from the system. Messaging protocols approved by the caBIG™ ARCHWS that are supported wherever messaging is indicated by the use cases.
Vocabularies/Terminologies and Ontologies	Terminologies reviewed and validated by the caBIG™ VCDEWS that are used for all appropriate data collection fields and attributes of data objects. Term definitions must meet VCDEWS workspace guidelines.
Data Elements	CDEs built from controlled terminologies and according to practices validated by the VCDEWS that are used throughout. CDEs are registered as ISO/IEC 11179 metadata components in the caBIG™ Context of the caDSR.
Information Models	Object-oriented domain information models are expressed in UML as class diagrams and as XMI files, and are reviewed and validated by VCDEWS.

**Abbreviations:** API, application programming interfaces; caBIG™, Cancer Biomedical Informatics Grid™; ARCHWS, Architecture workspace; VCDEWS, Vocabulary/Common Data Elements Workspace; CDE, Common Data Elements; ISO, International Organization for Standardization; IEC, International Electrotechnical Commission; caDSR, cancer Data Standards Repository; UML, Unified Modeling Language; XMI, XML Metadata Interchange.

**Table 3.** caBIG™ tools, infrastructure and data resources.

Category	Role
Tools	caIntegrator: a novel translational informatics platform that allows researchers and bioinformaticians to access and analyze clinical and experimental data across multiple clinical trials and studies.
	caBIO: a domain model and architecture used to model the rapidly-changing genomics and proteomics domain and to integrate data from numerous sources providing a holistic view of the human and mouse genomes.
	caTissue Core: a web tissue bank repository tool for biospecimen inventory, tracking, and basic annotation
	caArray: a web and programmatically accessible array data management system
	caXchange: a lab integration hub for clinical trials
	C3PR: a web patient registration system for clinical trials
Infrastructure	caGrid: enables universal mechanisms for providing interoperable programmatic access to data and analytics in caBIG™, creates a self-described infrastructure wherein the structure and semantics of data can be programmatically determined, and provides a powerful means by which services available in caBIG™ can be programmatically discovered and leveraged
	BRIDG: provides a shared view of the dynamic and static semantics that collectively define a shared domain-of-interest
	CTODS: provides a single, unified set of APIs that can access clinical data from multiple data sources
	caBIO: facilitates the communication and integration of information from the various initiatives supported by caBIG™ and NCI
	caCORE: helps streamline the informatics development and providing a common data management and application development framework
	caDSR: stores and manages CDEs developed by caBIG™ participants and various NCI-sponsored organizations
	EVS: produces the NCI Thesaurus, Metathesaurus and provides NCI with licenses for MedDRA, SNOMED, ICD-O-3, and other proprietary vocabularies
	caCORE SDK: a set of tools that aid in the design and creation of a “caCORE-like” software system.
Data Resources	caArray: an open-source, web and programmatically accessible array data management system
	caBIO: a biomedical data system built using a model-driven approach to develop objects, data models middleware, vocabularies, and ontologies for biomedical research.
	Cancer Gene Data Curation Pilot: creates a database of associations between genes and diseases and genes and drug compounds derived from the biomedical literature.
	caIntegrator: provides a mechanism for integrating and aggregating biomedical research data and access to a variety of data types
	caMOD: provides information about animal models for human cancer to the public research community
	Pathway Interaction Database: a highly structured, curated collection of information about known biomolecular interactions and key cellular processes assembled into signaling pathways

**Source:** https://cabig.nci.nih.gov/inventory/.

**Table 4.** The key caCORE components.

Component	Description
EVS	A description-logic based thesaurus and ontology management system. It is a set of services and resources that address NCI’s needs for controlled vocabulary.
caDSR	A repository that the NCI and its partners use it to create, edit and deploy the Common Data Elements.
caBIO	A model driven information system using the cancer Common Ontological Representation Environment; a synthesis of software, vocabulary, and metadata models for cancer research. Each of the caBIO domain objects represents an entity found in biomedical research such as Gene, Chromosome, Single Nucleotide Polymorphisms.
CSM	A comprehensive and integrated solution to common security objectives. It helps eliminate the need for development teams to create their own security methodology.

**Abbreviations:** EVS, Enterprise Vocabulary System; caDSR, cancer Data Standards Repository; caBIO, cancer Bioinformatics Infrastructure Objects; CSM, Common Security Module.

**Table 5A.** Queries used to identify NHL cases within linked legacy databases.

Query	Source	Criteria			Records Identified (% of all records)		
		FL	DLBCL	MCL	FL	DLBCL	MCL
Q1	Cancer Registry, ICD-O morphology codes plus behavior code 3	Morphology codes 9690, 9691, 9695, 9698	Morphology codes 9680, 9684	Morphology codes 9693	258 (40%)	337 (38%)	66 (8%)
Q2	Text search-pathology reports	‘follicular lymphoma’	‘diffuse large B’ and ‘diffuse large cell’	‘lymphoma lymphocytic’ OR ‘lymphocytic diffuse’ OR ‘lymphocytic lymphoma’	441 (69%)	367 (41%)	710 (82%)
Q3	Text search-pathology reports	‘follicular lymphoma’ OR ‘nodular lymphoma’ OR ‘Brill-Symmers’ OR ‘reticulosarcoma— follicular’ OR ‘follicular lymphosarcoma’ OR ‘follicle center lymphoma’ OR ‘follicular non-Hodgkin’	‘lymphoma diffuse histiocytic’ OR ‘lymphoma diffuse large’ OR ‘lymphoma large B’ OR ‘lymphoma large cell’ OR ‘lymphoma diffuse’ OR ‘diffuse non Hodgkin’ OR ‘lymphoma histiocytic’	‘mantle cell lymphoma’ OR ‘lymphoma mantle cell’ OR ‘mantle zone lymphoma’ OR ‘lymphoma mantle zone’	176 (27%)	321 (36%)	456 (52%)
Q4	Text search-all medical reports	Same as Q2	Same as Q2	Same as Q2	591 (92%)	567 (64%)	775 (89%)
Q5	Text search-all medical reports	Same as Q3	Same as Q3	Same as Q3	272 (42%)	471 (53%)	494 (57%)
	Total cases reviewed				643	886	871

**Abbreviations:** FL, Follicular lymphoma; DLBCL, Diffuse large B–cell lymphoma; MCL, Mantle cell lymphoma.

**Table 5B.** Sensitivity and specificity for linked database queries.

	Follicular Lymphoma		Diffuse Large B–cell Lymphoma		Mantle Cell Lymphoma	
QUERY	Sensitivity (%)	Specificity (%)	Sensitivity (%)	Specificity (%)	Sensitivity (%)	Specificity (%)
**Q1**	**53**	**73**	**65**	**60**	**54**	**98**
**Q2**	**70**	**33**	**38**	**66**	**72**	**17**
**Q3**	**37**	**82**	**55**	**78**	**85**	**51**
**Q4**	**97**	**13**	**55**	**50**	**44**	**6**
**Q5**	**57**	**73**	**78**	**56**	**97**	**48**

**Table 6.** Phase 1/Phase 2 Lymphoma clinical trial data collection schedule.

Procedures	Screen	Cycle 1–8 day 1	Cycle 1–8 day 8	End of cycle 2,4,6,8	End of induction	Every 3 months maintenance
Physical exam	X	X		X	X	X
ECG	X					
ECOG Performance Status	X	X	X	X	X	X
Chemotherapy administration	X					
Bortezomib administration		X	X			
Adverse Event assessment		X	X	X	X	X
Tumor assessment						
Chest, Abdomen, Pelvis CT scan	X			X	X	X
Pathology slides	X					
Tumor tissue block	X					
Bone Marrow Biopsy	X			X (if in remission)	X (if in remission)	X (if in remission)
Minimal Residual Disease Analysis	X			X (if in remission)	X (if in remission)	X (if in remission)
**Labs**						
Hematology (CBC)	X	X	X	X	X	X
Clinical Chemistry	X	X	X	X	X	X
(basic metabolic)						
Clinical Chemistry (hepatic)	X	X				
Uric Acid	X	X				
LDH	X					
Serum HCG	X					
HIV–1	X					
Hepatitis B	X					
Hepatitis C	X					
